# Comparative Study of Antennal Sensilla of Corixidae and Micronectidae (Hemiptera: Heteroptera: Nepomorpha: Corixoidea)

**DOI:** 10.3390/insects11110734

**Published:** 2020-10-27

**Authors:** Agnieszka Nowińska, Ping-ping Chen, Jolanta Brożek

**Affiliations:** 1 Institute of Biology, Biotechnology and Environmental Protection, University of Silesia in Katowice, Bankowa 9, 40-007 Katowice, Poland; agnieszka.nowinska@us.edu.pl; 2 Naturalis Biodiversity Center, P.O. Box 9517, NL-2300 RA Leiden, The Netherlands; pingping.chen@naturalis.nl

**Keywords:** Nepomorpha, Corixoidea, antennae, sensilla, morphology

## Abstract

**Simple Summary:**

Insects’ antennae are an important location for the structures responsible for sensorial perception. These structures have different morphologies and respond to different mechanical and chemical stimuli. Corixidae and Micronectidae are groups of insects living in water. Because of that, their antennae are much smaller than the antennae of terrestrial insects. Apart from that, their position on the evolutionary tree has been changed over the years. We analyzed the antennae of different species of Corixidae and Micronectidae and found five different types of sensillar structures. Moreover, by comparing the two groups together and with other water insects, we established similarities and differences when it comes to the types and distributions of these structures. These findings allowed us to support the current systematic position of the studied insects.

**Abstract:**

The goal of this study was to analyze the types and distributional patterns of sensilla in Corixoidea, which is part of the approach to the phylogeny study of Nepomorpha, based on the morphological characters of sensilla. This paper presents the results of the study, with the use of a scanning electron microscope (SEM), on the antennae of species from the families Corixidae and Micronectidae. The antennal sensilla of eleven species from Corixidae and two species from Micronectidae were studied. Five main types of sensilla with several subtypes of sensilla trichodea were found and described. The study has shown that the family Corixidae has a strong uniformity when it comes to antennal sensilla (similar patterns of sensilla trichodea and basiconica), and a similarity to the types and distributions of sensilla in two species of the family Micronectidae. However, significant differences between the families were also discovered (differences in sensilla presence on the first and second antennomeres, lack of sensilla coeloconica on the third antennomere in Micronectidae), which leads to a supportive conclusion of the systematic position of Micronectidae as a family.

## 1. Introduction

The superfamily Corixoidea includes 35 genera and about 590 species, according to a 2005 report by Chen et al. [1].

In 1988, Štys and Jansson [2] distinguished only Corixidae as one of 11 families within Nepomorpha (Nepidae, Belostomatidae, Corixidae, Aphelocheiridae, Potamocoridae, Naucoridae, Ochteridae, Gelastocoridae, Notonectidae, Pleidae, and Helotrephidae). In 2002, however, Nieser [3] increased the number of families within Corixoidea by upgrading Diaprepocorinae and Micronectinae to family status, according to characteristic features, such as, e.g., exposition or coverage of scutellum, differences in the build of the male paramere, and differences in the number of antennal segments or dissimilarity in stridulation. This change has been accepted and supported by the majority of other scientists [1,4,5,6,7].

Generally, the species of Corixoidea are small to medium sized specimens (0.5–15 mm), and dorsoventrally flattened. They usually have long hind legs with well-developed fringes of swimming hairs [1]. A basic morphological study of Corixidae was done by Jansson in 1986 [8]. They are usually regarded as organic scrapers [9,10]. However, in 2017 Hädicke et al. [11] distinguished three different feeding behavior patterns in Corixidae: ambush predators, who wait for prey attached to water plants (*Cymatia* sp. (Flor, 1860)); pelagic predators, who swim actively hunting for hyponeuston and free swimming prey (e.g., *Glaenocorisa* sp. Thomson, 1869); benthic omnivores, who collect food from the benthos while attached to the bottom of water reservoirs, with the occasional predation and grazing (e.g., *Sigara* sp. Fabricius, 1775 and *Micronecta* sp. Kirkaldy, 1897).

The general morphology of the family of micronectids has been studied by different authors in recent publications, such as Nieser [3], Nieser and Chen [5], Tinerella [6] and Chen et al. [12]. Considerable differences between corixids and micronectids were also pointed out by Larsén [13] and Nieser [3]. Larsén studied the genitalia of *Micronecta minutissima* (Linnaeus 1758) and *Corixa dentipes* (Thomson, 1869). He brought up differences in their male genital structures and sperm transmission.

A significant morphological difference between the families of Corixoidea regards the number of antennal segments. Four-segmented antennae are characteristic for Corixidae and Diaprepocoridae, but one to three-segmented antennae occur in Micronectidae [1,3].

In this study, we attempt to find the differences between Corixidae and Micronectidae in terms of antennal sensilla, with the expectation of finding significant characters that reinforce their systematic/phylogenetic position.

In the early stages of phylogenetic studies, Corixidae (with Micronectinae and Diaprepocorinae as subfamilies) had been treated as a basal/sister group to the rest of nepomorphan families [14,15]. According to China [14], it was due to being the sole, not-fully predaceous group, derived from phytophagous ancestors, in contrast to the rest of aquatic bugs who much later arose from predaceous ancestors.

Later studies done by China (in 1955) [16], Popov (in 1971) [17], Mahner (in 1993) [18], Hebsgaard et al. (in 2004) [19], Brożek (2014) [20], and the recent 2019 study by Ye et al. [21], placed Corixidae/Corixoidea as a second branch of the phylogenetic tree, after the clade Nepidae + Belostomatidae. Nevertheless, in 1967, Rieger [22] regarded Corixidae as a more evolutionarily advanced group and placed it as a third branch, while the second branch was occupied by the clade Ochteridae + Gelastocoridae. The results of Hua et al. [23] from 2009 showed Corixoidea as the basal clade—a sister group—to the remaining clades of Nepomorpha. Similarly, a study by Wang et al. [24] in 2015 indicated Micronectidae and Corixidae as the first branches of Nepomorpha’s phylogenetic tree. More studies are necessary to establish the true positioning of the clades.

Insects are able to perceive different types of stimuli using specialized structures called sensilla. These structures are composed of auxiliary cells, which are modified epidermal cells and bipolar sensory neurons, which are surrounded by a dendrite sheath. Each sensillum possesses a specialized sensor in the cuticle, which captures the signal and converts it to be carried by sensory neurons to the brain. Mechano-, chemo-, and thermo-hygroreceptive sensilla are distinguished based on their internal and external structures. Sensilla that possess pores (multiporous, uniporous) are usually chemoreceptive sensilla, while sensilla with a surface without pores are mechano- or thermo-hygroreceptive sensilla [25,26,27,28,29,30,31].

In Nepomorpha, antennal sensilla were studied in Nepoidea (in 2019) [32], Aphelocheiridae, Ochteridae, and Gelastocoridae (in 2020) [33]. A few species (*Notonecta glauca* Linnaeus, 1758, *Plea minutissima* Leach, 1817, *Nepa cinerea* Linnaeus, 1758, *Ranatra linearis* (Linnaeus, 1758), and *Sigara striata* (Fieber, 1848)) were also studied by Chaika and Sinitsina [34] in 1999. According to the latter authors, the antennae of Nepomorpha are small and mostly hidden beneath the head. They hypothesize that this arrangement led to sensilla being located mainly on one side of the antennae, because the dorsal side of Nepomorphan’s antennae (which is the closest to the head) is generally devoid of sensilla or has them in very small numbers [34]. They also described some basic types of antennal sensilla.

By examining the same species studied by Chaika and Sinitsina [34], with the use of scanning electron microscopy, Nowińska and Brożek [32] added extra antennal sensillar types to the results of the former authors, which revealed a greater variety of sensilla types on antennae.

Knowledge on the antennal sensilla of Corixoidea is poor. Therefore, the aim of this study is to explore the morphological types and distributions of the antennal sensilla of Corixidae and Micronectidae, in order to evaluate their current systematic position, and to compare them with other nepomorphan families that have already been studied.

## 2. Materials and Methods

The materials were obtained from the collections of the National History Museum in Prague, the Zoological Museum of the State Moscow University and the Hungarian Natural History Museum in Budapest. All specimens were cleaned in an ultrasound cleaner, the antennae were dissected, dried in ethanol, mounted, sputtered with gold or chromium, and observed with the use of the scanning electron microscopes Phenom XL and Hitachi UHR FE-SEM SU 8010 in the scanning microscopy laboratory of the Faculty of Natural Science, Institute of Biology, Biotechnology and Environmental Protection of Silesian University in Katowice. The specimens obtained from the Hungarian Natural History Museum in Budapest (Hungary) and their dissected antennae are currently stored in the University of Silesia in Katowice, Institute of Biology, Biotechnology and Environmental Protection (Poland). However, they are to ultimately be sent back and stored in the Museum. The specimens (along with the dissected antennae) provided by the National History Museum in Prague (Czechia) and the Zoological Museum of the State Moscow University (Russia), are currently stored in the University of Silesia in Katowice, Institute of Biology, Biotechnology and Environmental Protection (Poland).

We follow the terminology and classification reported in other papers on antennal sensilla of insects [26,32]. The antennae of thirteen species from five subfamilies were studied (Table 1).

## 3. Results

**Figure 1:**
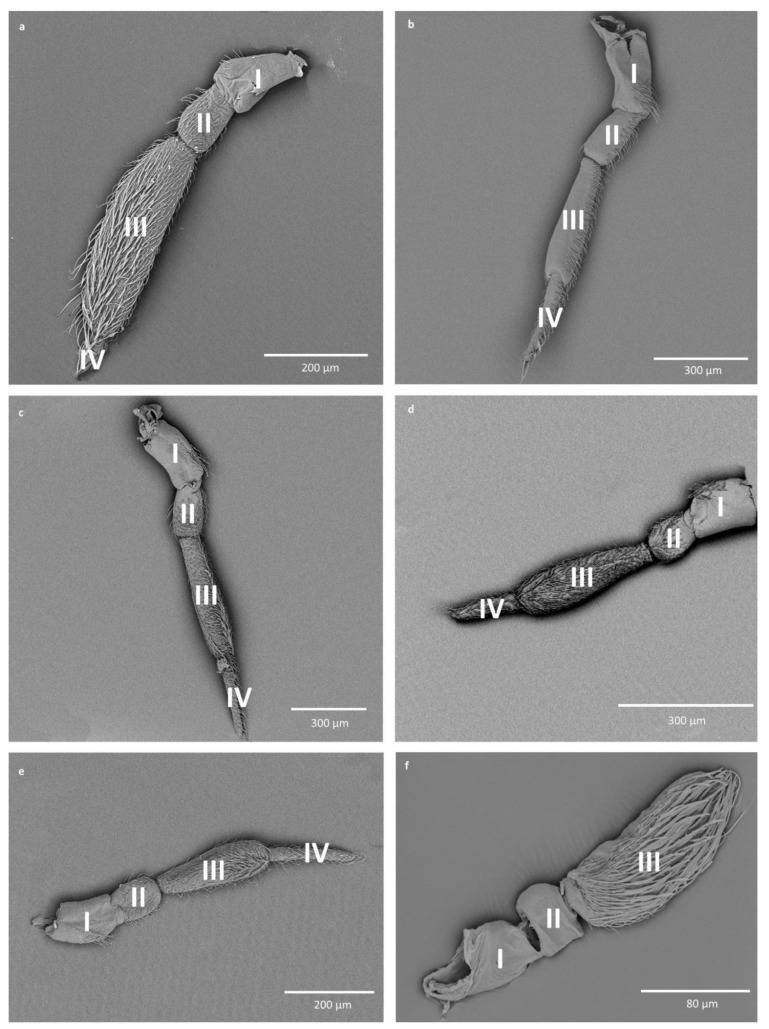
Shapes and sizes of the antennae of Corixoidea. (**a**) *Agraptocorixa hirtifrons*; (**b**) *Corixa affinis*; (**c**) *Corixa punctata*; (**d**) *Paracorixa concinna*; (**e**) *Sigara striata*; (**f**) *Micronecta poweri*; (I–IV) numbers of the antennomeres.

We observed that the antennae of Corixidae differ slightly in shape and size among its species, especially when it comes to the length of the fourth antennomere (Figure 1a–e). However, the antennae of Micronectidae showed great differences from Corixidae’s antennae, not only in shape and size but also in the number of antennal segments (Figure 1f).

**Figure 2:**
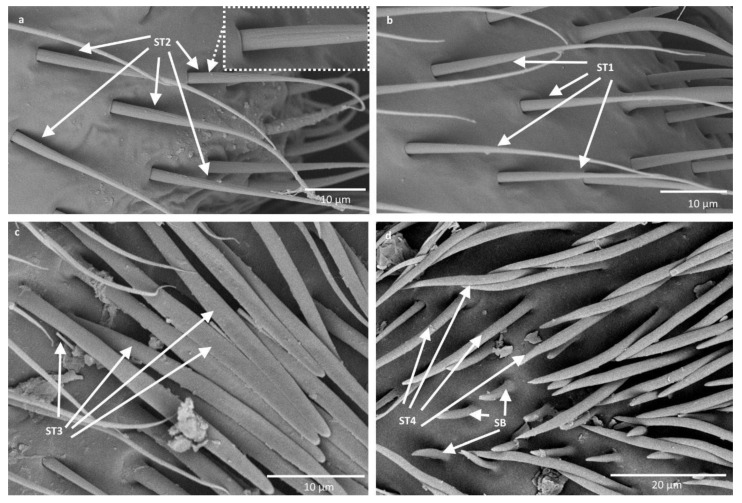
Antennal sensilla of Corixoidea. (**a**,**b**) *Corixa punctata*; (**c**) *Sigara striata*; (**d**) *Callicorixa praeusta*. ST—sensilla trichodea; SB—sensilla basiconica.

**Figure 3:**
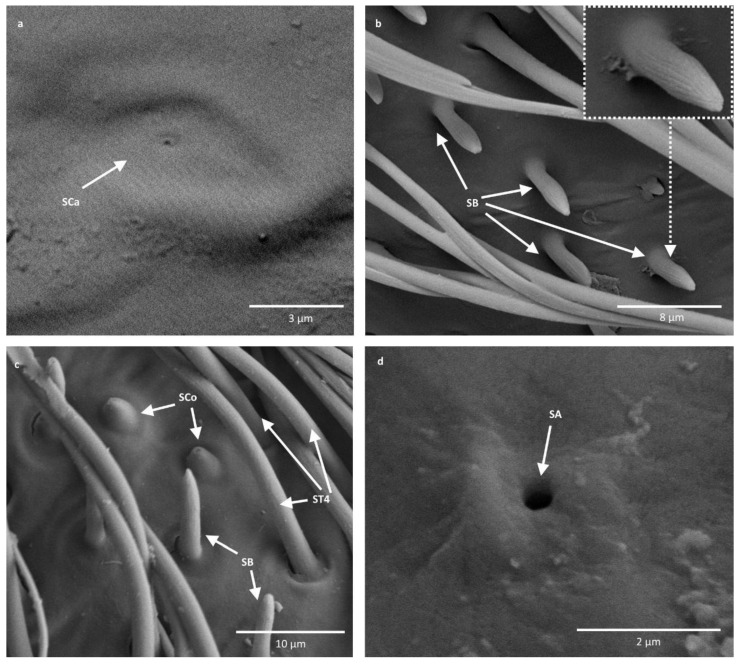
Antennal sensilla of Corixoidea. (**a**) *Paracorixa concinna*; (**b**) *Cymatia coleoptrata*; (**c**) *Corixa dentipes*; (**d**) *Stenocorixa protrusa*. SCa—sensilla campaniformia; SB—sensilla basiconica; SCo—sensilla coeloconica; ST—sensilla trichodea; SA—sensilla ampullacea.

We recognized five main types of sensilla on the surface of the antennae of all studied species of Corixidae and Micronectidae (Table 2):

1. Sensilla trichodea—sensilla responsible for mechanoreception: straight, long and hair like structures. Usually they cover a big surface area on the antennae. They rise from flexible sockets, which is common for mechanoreceptive sensilla. A flexible socket is a socket with a thin cuticular membrane that is continuous with the general body’s cuticle and hair. Flexible sockets provide greater mobility at the base of sensilla. Among sensilla trichodea, the following subtypes were observed:

- Sensilla trichodea (ST1)—straight hair like structures with a smooth surface and pointed tip. They generally occur in large numbers on the surface of antennae and were found in all studied species (Figure 2b).
- Sensilla trichodea (ST2)—straight structures with a pointed tip and ribbed surface. This subtype occurs often on insects’ antennae and was found in all studied species (Figure 2a).
- Sensilla trichodea (ST3)—round at the base and ribbed structures, which flatten from the middle of the length, resembling a leaf-like structure. These sensilla were found in *Agraptocorixa hirtifrons, Sigara nigrolineata, Sigara striata, Micronecta poweri,* and *Micronecta scholtzi* (Figure 2c).
- Sensilla trichodea (ST4)—straight, thick structures with rounded or pointed tip and ribbed surface. These sensilla were found in all studied species except *Agraptocorixa hirtifrons* (Figure 2d and Figure 3c).

It is significant that ST1 and ST2 are classic sensilla trichodea because of their hair-like build, whereas ST3 and ST4 derived from these structures, presenting a slightly changed model.

2. Sensilla campaniformia (SCa)—sensilla also responsible for mechanoreception, therefore rising from flexible sockets. These are flat structures, resembling round or oval discs, with a single pore in the middle. These sensilla were found in *Paracorixa concinna* and *Sigara nigrolineata* (Figure 3a).

3. Sensilla basiconica (SB)—sensilla responsible for chemoreception, usually olfaction. Their surface is covered with wall pores, which allow chemical particles to enter. Unlike mechanoreceptive sensilla, these structures arise from inflexible sockets, so without the membrane that allows greater mobility at the base of the sensillum. The sensilla basiconica present on the antennae of Corixoidea are smooth at the base and ribbed along the length. They were found in all studied species (Figure 2d and Figure 3c).

4. Sensilla coeloconica (SCo)—sensilla responsible for thermo-hygroreception. They are peg-like structures embedded in a cavity of the cuticle, in inflexible sockets. They have a nonporous surface but there is one pore present at the tip of the sensillum. These sensilla were found in all the species of the family Corixidae (Figure 3c).

5. Sensilla ampullacea (SA)—thermo-hygroreceptive sensilla, embedded in a deep cavity which makes them invisible from the outside. They are small pegs arising from inflexible sockets. Ultrastructural studies were not done on these structures; therefore, their classification is tentative. However, taking into account their morphological appearance, and comparing it with other possibilities (glandular structures, which reportedly should form a sieve structure, rather than appear singularly), we assigned these structures to this type of sensilla (Figure 3d).

**Figure 4:**
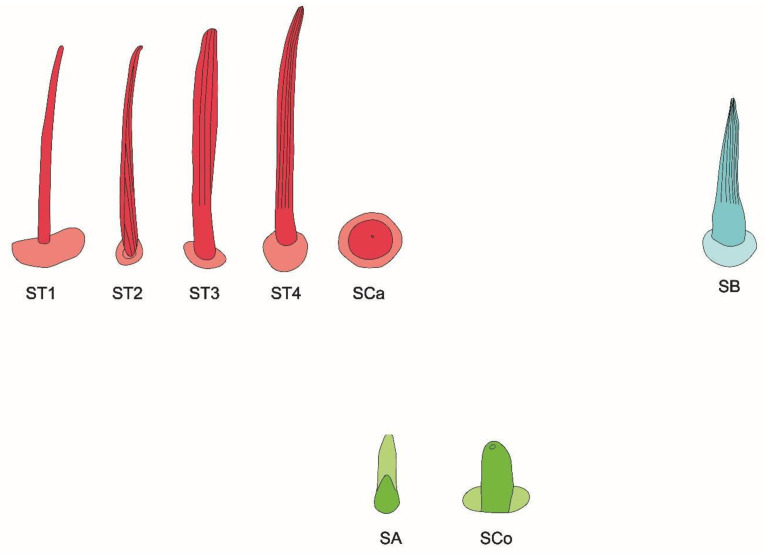
Schematic of antennal sensilla. ST—sensilla trichodea; SCa—sensilla campaniformia; SB—sensilla basiconica; SA—sensilla ampullacea; SCo—sensilla coeloconica.

The schematic of antennal sensilla is provided in Figure 4.

## 4. Discussion

This paper contains a set of morphological studies on the antennal sensilla of species of the superfamily Corixoidea (Nepomorpha). The antennal sensilla of 12 species were analyzed with the use of a scanning electron microscope.

Until now, only the labial sensilla of Corixidae have been studied [35]. Sensilla on the labium are represented by chaetica, peg, papillae, and ribbon-like forms.

The types of sensilla found in the studied species represent three different methods of gathering information from the environment—mechanoreception, chemoreception, and thermo-hygroreception. In water, these structures play very important roles.

Mechanoreceptive sensilla can detect vibrations from sound or movement, which are carried further than in the atmosphere, due to the water’s density. Moreover, considering that the water medium is not ideal when it comes to visibility, the role of mechanosensilla in orientation becomes even more important [25].

Chemoreceptive sensilla help with the detection of small chemicals dissolved in water. They help in intra- and inter-specific relationships, such as predator avoidance or foraging [36,37]. The chemosensilla found in our studies were multiporous, which indicates an olfactory function.

As for thermo-hygroreceptive sensilla, they are mostly important for terrestrial animals, which need to control their water balance and sense humidity and temperature variations. Rebora et al. [22] suggest that hygroreception in organisms living in water is redundant. However, other studies on water insects [32,33,38] prove the existence of these structures on the antennae of such insects. Thermo-hygroreceptive sensilla show their relevance, e.g., during the migrations of water insects, which have also been observed in Corixidae [39]. Thermo-hygroreceptive sensilla play a role in finding new water bodies. In this study, we found such sensilla in all of the species from the family Corixidae.

The representatives of the family Corixidae display a pattern when it comes to the presence of sensilla types and their distribution (Table 2). The occurrence of sensilla trichodea, basiconica, and coeloconica was observed in all the species from these subfamilies. Moreover, the same types were found on the same antennomeres in all the species. The first, second and fourth antennomeres are covered mainly with sensilla trichodea (ST1 and ST2), with rare occurrences of a singular sensilla basiconica (SB) and/or coeloconica (SCo) on the second or fourth antennomere. Subtypes of sensilla trichodea (ST2, ST3/ST4), sensilla coeloconica (SCo), and sensilla basiconica (SB) were found on the third antennomere. Notably, sensilla basiconica and coeloconica occur in large numbers on this antennomere, usually aligned with the length of the antennomere. Sensilla trichodea (ST3/ST4) create a form of shield for shorter sensilla, as they bend towards the antennomere, covering the surface of the antennomere. Sensilla campaniformia and ampullacea occur rarely in general, and when they do occur, they do so on different antennomeres. In other words, there is no apparent pattern when it comes to the occurrence of sensilla campaniformia and ampullacea.

Other studies on the antennal sensilla of insects suggest that sensilla basiconica and sensilla coeloconica play a role in detecting the odors of host plants and this crucial function is performed especially by the sensilla on the last antennomeres [40,41,42].

According to our knowledge, corixids are mainly detritivores (only a few are predators), which might explain the characteristic distribution of their antennae’s chemoreceptive sensilla. Compared to the previously studied nepomorphan groups [32,33], Corixidae is the first family that displays a great uniformity in the arrangement of the antennal sensilla of its species. Not all of the genera of Corixidae were studied. However, based on the obtained results and in comparison to other studied taxa, Corixidae displayed the most similarities within the group. The sensilla coeloconica and basiconica in other nepomorphan taxa are rarely arranged in groups and never on the same antennomere in all of the studied species of each taxon. It may indicate a connection between the arrangement of sensilla and diets, as well as Corixidae’s systematic position. However, a confirmation of this hypothesis would need a comprehensive comparative analysis of all the families of Nepomorpha.

In their studies, Chaika and Sinitsina [34] claimed that Nepomorpha’s antennae size reduction led to transferring the antennal sensilla to the external side of the antennae, and also that all of the antennae of Nepomorpha are hidden near the head. From later studies [33], we know that in Aphelocheiridae and Ochteridae, for example, the antennae are well visible and not placed strictly close to the head. Moreover, the antennal sensilla of the families Gelastocoridae and Ochteridae are evenly distributed throughout all sides of the antennae. However, it is worth mentioning that the families Ochteridae and Gelastocoridae are considered semiaquatic, or even terrestrial insects. Therefore, differences between their morphology and the morphology of other fully aquatic Nepomorpha are expected. Chaika and Sinitsina [34] mentioned that active movers, such as *Sigara* sp., have better developed antennal sensilla, especially on the two middle antennomeres, which might be responsible for the detection of water fluctuations. In this study, we observed numerous sensilla on all of the antennomeres.

Chaika and Sinitsina [34] have only studied one species from the family Corixidae—*Sigara striata*—which is also included in our study. According to Chaika and Sinitsina [34], sensilla in this species are concentrated on one side of the antennae (the majority of which are sensilla trichodea of two types—pointed and blunt at the tip), distal antennomeres are thinner than basal ones, and all of the sensilla (trichodea and basiconica) are bent towards the end of the antennae.

Our studies confirm that the antennal sensilla are concentrated on one side of the antennae and that are mostly sensilla trichodea. However, we distinguished not two, but four subtypes of these sensilla (ST1, ST2, ST3, and ST4), which include flattened sensilla trichodea (ST3) (Figure 2c and Figure 4). Additionally, the types of sensilla trichodea across different antennomeres are not identical. The first, second, and fourth antennomeres are covered with two subtypes of sensilla trichodea (ST1 and ST2), while on the third antennomere we observed three subtypes (ST2, ST3, and ST4). Chaika and Sinitsina [34] noted the existence of sensilla basiconica, which we also found. However, these authors did not mention other types of sensilla, while we documented the presence of sensilla campaniformia, ampullacea, as well as numerous sensilla coeloconica.

Regarding the differences in the width of antennomeres: the two basal antennomeres are of more or less the same width, the third antennomere is slightly narrower at the base than the first two antennomeres, and the last antennomere is considerably narrower than all the other antennomeres (Figure 1).

Unfortunately, we did not have access to material of the subfamily Synaptogobinae (a newly described subfamily of Micronectidae [5]). Differences between their antennae and the antennae of other Micronectidae species have been found. In Synaptogobinae, antennae have only one segment, and are fused with the head capsule. Nieser and Chen [5] also described very fine hairs, when compared to other genera of Micronectidae. It would be interesting to see the morphological differences of Synaptogobinae’s antennal sensilla in comparison to the other species of the family.

The antennal sensilla of three subfamilies of Corixidae were studied. Differences in antennal sensilla between the subfamilies of Nepoidea have been reported in previous studies [32]. Therefore, differences between the subfamilies of Corixidae were expected. However, no significant dissimilarities were noted.

On the other hand, we observed differences between Micronectidae and Corixidae (Table 2). The antennae are three-segmented in Micronectinae (the subfamily Synaptogobinae has only one antennal segment, however). We did not observe any sensilla on the first and second antennal segments in *Micronecta* (Micronectinae). The characteristic pattern (with sensilla basiconica and trichodea, observed in Corixidae’s species on the second to last antennomere) was also observed in *Micronecta* but on the last antennomere. On the micronectids’ last antennomere, we observed sensilla basiconica covered by sensilla trichodea ST3, as well as other subtypes of sensilla trichodea (ST1, ST4) on the edges of the antennomere. This arrangement resembles the one on the third antennomere in the corixids, lacking only sensilla coeloconica. We believe that thermo-hygrosensilla are also present in *Micronecta*. However, the small size of the antennae of these species (around 250–300 µm) makes the antennal sensilla also smaller in size and very difficult to spot. Our study confirms the close relation between these two families (similar patterns of sensilla trichodea and basiconica, which have not been found in other studied families of Nepomorpha), and is supportive of their present systematic position as two separate families (due to differences in sensilla presence on the first and second antennomeres, lack of sensilla coeloconica on the third antennomere in Micronectidae).

In comparison to previously studied nepomorphan families, common types of sensilla might be established. In the superfamily Nepoidea, five common types with Corixoidea (ST1, ST2, SCa, SA, and SCo3, which resembles SCo in this paper) have been found [32]. In the superfamily Ochteroidea, five common types (ST1, ST2, SB4, which resembles SB described in this paper, SA and SCo) have been described. The least common types with Corixoidea have been documented in the family Aphelocheiridae (ST2, SCa, and SA) [33].

Moreover, Corixidae’s ST4 resembles the sensilla chaetica present in most of the previously studied groups. However, the differences between these two sensilla do not allow us to consider these two types as identical. By definition, sensilla chaetica are rigid [30], whereas sensilla trichodea ST4 are always bent toward the end of the antennae, covering the sensilla underneath.

In summation, the sensilla present on the antennae of the families Corixidae and Micronectidae share common types with each other, and also with every other nepomorphan family studied to date. Previous studies on the antennal sensilla of Nepoidea [32], which took into account previous phylogenetic studies by other authors which placed Nepoidea and Corixoidea as a sister group to other nepomorphan families, seem to reinforce the placement of this superfamily as a sister group. Those studies showed a great variety of mechanoreceptive sensilla in Nepoidea, which have, so far, not been found in any other studied nepomorphan taxa.

The current study not only reinforces the findings of Nieser [3] treating the studied taxa as two separate families, but also found a specific set of sensilla characteristic for Corixoidea (ST1, ST4, SB, SCo), which is always present on the third antennomere and is an autapomorphic feature for this taxon, which supports the phylogenetic position suggested by Ye et al. [21].

## 5. Conclusions

Based on the presently studied species, Corixidae displays the highest uniformity in terms of sensilla types and their distribution when compared to all the nepomorphan families studied before. This differentiates the family from all other nepomorphan families and shows close relations among species within the family. The family Micronectidae differs from Corixidae mainly in the number of antennomeres (three or one, not four as in Corixidae). They do, however, display a similar distribution pattern of olfactory sensilla. A characteristic pattern of sensilla was discovered and described on the last antennomere in Micronectidae and the third antennomere of Corixidae. Although Micronectidae shares certain similarities with Corixidae, like the same types of sensilla trichodea and basiconica, and a similar pattern of these sensilla on the third antennomere, there are still significant differences between these two families in terms of antennal sensilla, like the lack of sensilla on the first two antennomeres in Micronectidae. This is one of the distinguishing features of Micronectidae from the other two families in Corixoidea.

## Figures and Tables

**Table 1:** Studied material of Corixidae and Micronectidae.

Family	Subfamily	Tribe	Species	Institution that Supplied the Specimens	Label Data	Country Where the Specimens Were Collected
**Corixidae**	**Corixinae**	Agraptocorixini	*Agraptocorixa hirtifrons* (Hale, 1922)	Hungarian Natural History Museum (Hungary)	Vezényi; Asuncion, Villa Morra	Paraguay
		Corixini	*Callicorixa praeusta* (Fieber, 1848)	National History Museum in Prague (Czechia)	Starovice, rybnik	Czechia
			*Corixa affinis* Leach, 1817	National History Museum in Prague (Czechia)	or. Safranovo, shore	Bulgaria
			*Corixa dentipes* (Thomson, 1869)	National History Museum in Prague (Czechia)	CZ. 7052, Křemže, MŘÍČ, CK, Borský rybkin, zarostlý litorál sev. břehu	Czechia
			*Corixa punctata* (Illiger, 1807)	National History Museum in Prague (Czechia)	Ružbachy-Lacková	Slovakia
			*Paracorixa concinna* (Fieber, 1848)	National History Museum in Prague (Czechia)	Jindrychovice, KT, 6746, Rybnik u obce	Czechia
			*Sigara nigrolineata* (Fieber, 1848)	National History Museum in Prague (Czechia)	6874, Potecv, ZL, Rybnik u obce	Czechia
			*Sigara striata* (Linnaeus, 1758)	National History Museum in Prague (Czechia)	Albrechtičky, NJ, 6274, Lučni Tůne u Odry	Czechia
	**Cymatianinae**		*Cymatia coleoptrata* (Fabricius, 1777)	National History Museum in Prague (Czechia)	Slov. occid. Záhorie, Mor. Sv, Ján, Dlhé lúky	Slovakia
	**Stenocorixinae**		*Stenocorixa protrusa* Horváth, 1926	Zoological Museum of the State Moscow University (Russia)	Species identified in the Museum, the place of collection cannot be confirmed	Russia
**Micronectidae**			*Micronecta poweri* (Douglas and Scott, 1869)	National History Museum in Prague (Czechia)	CZ. Bohemia occ. 6046, Ondřejov, PS, Střela u sil. mostu Křečov-Miadotice	Czechia
			*Micronecta scholtzi* (Fieber, 1860)	National History Museum in Prague (Czechia)	Újezd u Brna, rybnicek u obce	Czechia

**Table 2:** The comparison of sensilla types present in the studied species. ST—sensilla trichodea; SCa—sensilla campaniformia; SB—sensilla basiconica; SA—sensilla ampullacea; SCo—sensilla coeloconica.

Species	Sensilla Present on the First Antennal Segment	Sensilla Present on the Second Antennal Segment	Sensilla Present on the Third Antennal Segment	Sensilla Present on the Fourth Antennal Segment
*Agraptocorixa hirtifrons*	ST1, ST2	ST1, ST2	ST2, ST3 SB SCo	ST1, ST2
*Callicorixa praeusta*	ST1, ST2	ST1, ST2 Single SB	ST2, ST4 SB SCo	ST1, ST2 Single SB
*Corixa affinis*	ST1, ST2	ST1, ST2 Single SB	ST2, ST4 SB SCo	ST1, ST2 Single SB
*Corixa dentipes*	ST1, ST2	ST1, ST2 Single SB SA	ST2, ST4 SB SCo	ST1, ST2 Single SCo
*Corixa punctata*	ST1, ST2	ST1, ST2	ST2, ST4 SB SCo	ST1, ST2
*Paracorixa concinna*	ST1, ST2 SCa	ST1, ST2	ST2, ST4 SB SCo	ST1, ST2
*Sigara nigrolineata*	ST1, ST2 SCa SA	ST1, ST2	ST2, ST3, ST4 SB SCo	ST1, ST2
*Sigara striata*	ST1, ST2 SCa	ST1, ST2	ST2, ST3, ST4 SB SCo, SA	ST1, ST2
*Cymatia coleoptrata*	ST1, ST2	ST1, ST2	ST2, ST4 SB SCo	ST1, ST2
*Stenocorixa protrusa*	ST1, ST2	ST1, ST2 Single SB SA	ST2, ST4 SB SCo, SA	ST1, ST2
*Micronecta poweri*	No sensilla documented	No sensilla documented	ST1, ST3 SB	-
*Micronecta scholtzi*	No sensilla documented	No sensilla documented	ST1, ST3, ST4 SB	-
